# Confronting Upside-Down Video-Assisted Thoracic Surgery for Anterior Mediastinal Leiomyoma

**DOI:** 10.70352/scrj.cr.25-0773

**Published:** 2026-03-26

**Authors:** Yuma Takeuchi, Tomonari Oki, Shuhei Iizuka, Yoshiro Otsuki, Toru Nakamura

**Affiliations:** 1 Department of General Thoracic Surgery, Seirei Hamamatsu General Hospital, Hamamatsu, Shizuoka, Japan; 2 Department of Pathology, Seirei Hamamatsu General Hospital, Hamamatsu, Shizuoka, Japan

**Keywords:** mediastinal neoplasms, leiomyoma, thoracic surgery, video-assisted

## Abstract

**INTRODUCTION:**

Leiomyoma is a benign smooth muscle tumor typically arising in the uterus or gastrointestinal tract; its occurrence in the anterior mediastinum is extremely rare. The differential diagnosis of anterior mediastinal tumors usually includes thymoma, germ cell tumors, and lymphoma, whereas leiomyoma is rarely considered.

**CASE PRESENTATION:**

A 50-year-old woman with no notable medical history presented with chest pain and cough. Chest radiography showed a mass in the left lower lung field. Tumor markers (HCG, CEA, AFP, CYFRA 21-1, ProGRP, anti-AchR antibody, sIL-2R) were normal. CT revealed a 2.8 × 1.8 × 2.3 cm solid mass in the left anterior mediastinum, and MRI showed a fibrous lesion with mildly high T2 signal intensity and gradual contrast enhancement. These findings suggested a solitary fibrous tumor or neurogenic tumor. Thoracoscopic surgery was performed using the confronting upside-down video-assisted thoracic surgery (VATS). Intraoperatively, a well-defined, whitish, pedunculated mass arising from pericardial fat was identified. En bloc resection of the mass and surrounding pericardial fat was completed, while the phrenic nerve was entirely preserved. Histopathological examination revealed spindle-shaped smooth muscle cell proliferation. Immunohistochemistry was positive for desmin and α-smooth muscle actin (αSMA), partially positive for CD34, and negative for S100, confirming leiomyoma. The postoperative course was uneventful, with discharge on day 2.

**CONCLUSIONS:**

Primary leiomyoma of the anterior mediastinum is extremely rare. Because primary mediastinal leiomyomas have nonspecific imaging features, distinguishing them from other anterior mediastinal tumors is challenging; thus, surgical resection is required for both diagnosis and treatment. Compared with the conventional look-up method, the confronting upside-down VATS technique utilizes higher intercostal access to provide a comprehensive thoracic view while minimizing diaphragmatic interference. The 180-degree rotated assistant monitor allows both surgeon and assistant to share a thoracotomy-like visual field, enhancing intraoperative coordination. In this case, it facilitated a stable operative field and phrenic nerve preservation despite the tumor’s proximity without diaphragmatic interference. The confronting upside-down VATS technique may be particularly advantageous for anterior mediastinal tumors located along the phrenic nerve and in close proximity to the diaphragm.

## Abbreviation

VATS
: video-assisted thoracic surgery

## INTRODUCTION

Leiomyoma is a benign neoplasm that typically arises in smooth muscle-containing organs such as the uterus and the gastrointestinal tract, with those arising in the anterior mediastinum being particularly uncommon. The differential diagnosis for anterior mediastinal tumors commonly includes thymoma, germ cell tumors, and lymphoma, while leiomyoma is rarely considered. In fact, even in large-scale cohort studies of mediastinal tumors, leiomyomas are so rare that they do not appear as a distinct category.^1,2)^ Primary mediastinal leiomyomas are usually discovered incidentally and remain asymptomatic,^3)^ although tumor enlargement could lead to compression of the adjacent organs, resulting in symptoms such as cough or dyspnea.^4–8)^

On imaging diagnosis, including CT and MRI, these tumors appear as well-defined masses. However, features such as homogeneous soft tissue density and mild contrast enhancement are nonspecific, and definitive diagnosis requires pathological evaluation of surgically resected specimens.

To date, mediastinal tumors have often been treated using median sternotomy, open thoracotomy, look-up VATS, or robot-assisted thoracoscopic surgery (RATS).^9–14)^ However, a confronting upside-down VATS approach has been rarely employed.

We herein report a rare case of a primary anterior mediastinal leiomyoma resected using the confronting upside-down VATS approach.

## CASE PRESENTATION

**Fig. 1:**
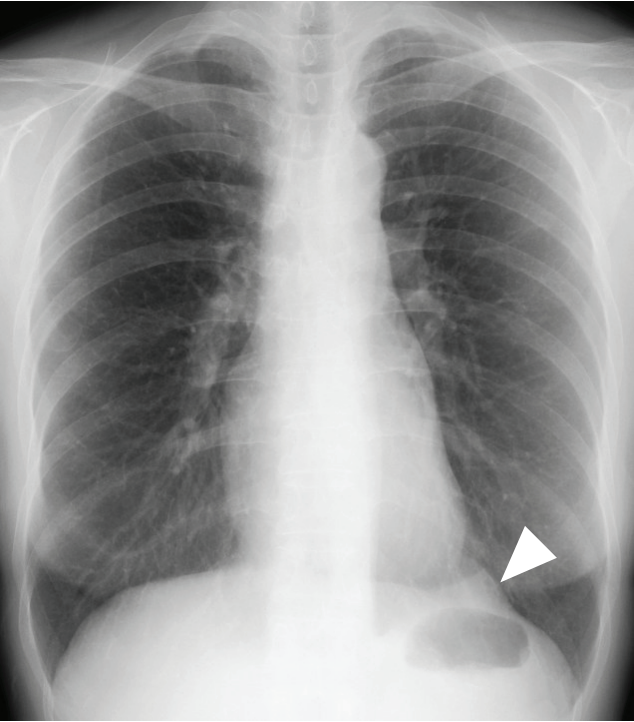
Chest radiograph showing a nodule along the pericardium toward the left hemidiaphragm (arrowhead).

**Fig. 2:**
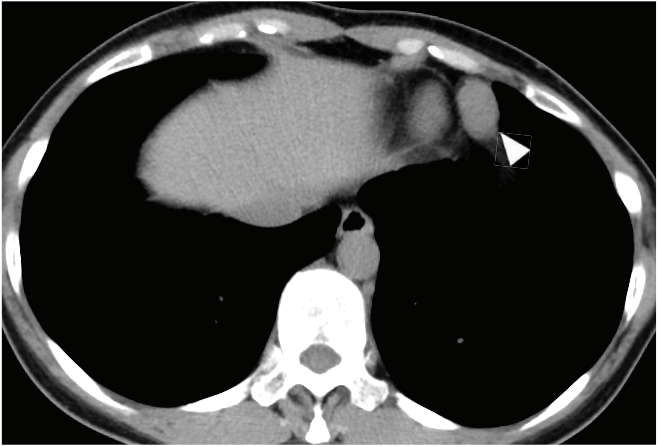
Plain axial CT image showing a solid mass in the left anterior mediastinum (arrowhead).

**Fig. 3:**
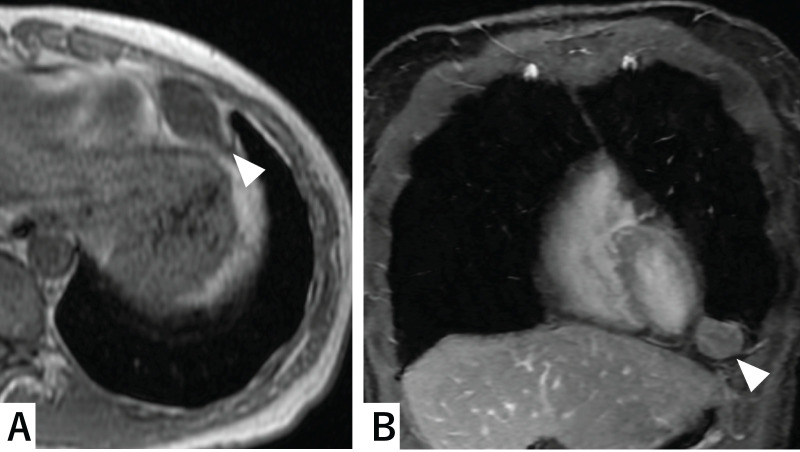
T2-weighted MRI showing a mildly high signal intensity (arrowheads). (**A**: axial, **B**: coronal).

A 50-year-old woman with no notable medical history, family history, or smoking history presented for evaluation of chest pain and cough. Chest radiography revealed a mass shadow in the left lower lung field (**Fig. 1**). Laboratory tests including human chorionic gonadotropin (HCG), carcinoembryonic antigen (CEA), alpha-fetoprotein (AFP), cytokeratin 19 fragment (CYFRA 21-1), pro-gastrin-releasing peptide (ProGRP), anti-acetylcholine receptor antibody (anti-AchR antibody), and soluble interleukin-2 receptor (sIL-2R) were all within normal limits. No other significant clinical findings were noted. Plain chest CT revealed a solid mass measuring 2.8 × 1.8 × 2.3 cm in the left anterior mediastinum (**Fig. 2**). MRI suggested a fibrous, solid mass exhibiting a mildly high signal intensity on T2-weighted images and slowing gradual contrast enhancement (**Fig. 3A** and **3B**). No additional masses or abnormalities were identified elsewhere. Based on these imaging findings, a solitary fibrous tumor or neurogenic tumor was suspected, and surgical resection was planned.

**Fig. 4:**
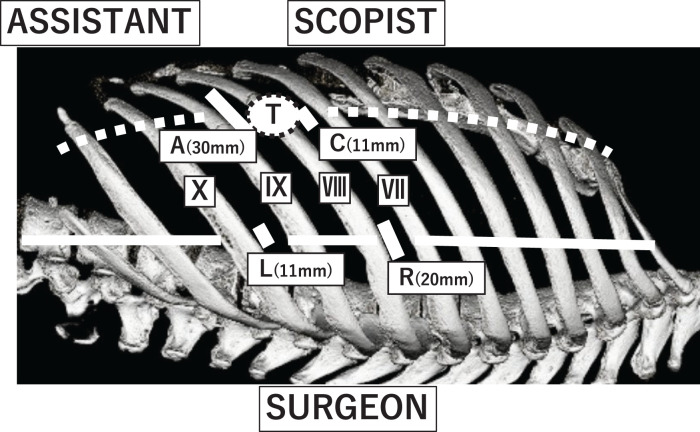
Port insertion sites: a 2-cm utility port for the surgeon’s right hand (R) placed in the 7th ICS along the posterior axillary line; a 11-mm port for the surgeon’s left hand (L) in the 9th ICS along the posterior axillary line; an 11-mm camera port (C) in the 7th ICS along the anterior axillary line; and a 3-cm utility port for the assistant (A) along the anterior axillary line. The approximate location of the tumor (T) is as shown. The anterior axillary line is indicated by a dotted line, whereas the posterior axillary line is indicated by a solid line.

**Fig. 5:**
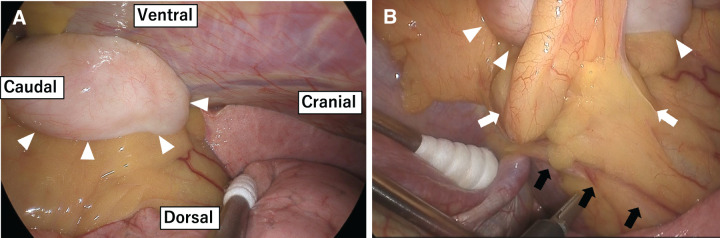
(**A**) A well-defined, whitish lesion approximately 2.5 cm was identified, pedunculated from the pericardial fat (arrow heads). (**B**) The tumor (arrowheads) and the surrounding pericardial fat (white arrows) were resected en bloc, while the phrenic nerve (black arrows) was preserved. A sponge stick is used to retract the diaphragm caudally.

Thoracoscopic surgery was performed using a confronting upside-down monitor setting. An 11-mm camera port was placed at the anterior axillary line of the 7th intercostal space (ICS), another 11-mm port for the surgeon’s left hand at the anterior axillary line of the 9th ICS, a 2-cm utility port for the surgeon’s right hand at the posterior axillary line of the 7th ICS, and a 3-cm utility port for the assistant at the anterior axillary line of the 8th ICS (**Fig. 4**). Intraoperatively, a well-defined, whitish mass approximately 2.5 cm was identified, pedunculated from the pericardial fat (**Fig. 5A**). No pleural effusion was observed. The phrenic nerve was identified along its entire course and preserved at an adequate margin from the tumor (**Fig. 5B**). En bloc resection of the tumor and surrounding pericardial fat was performed.

**Fig. 6:**
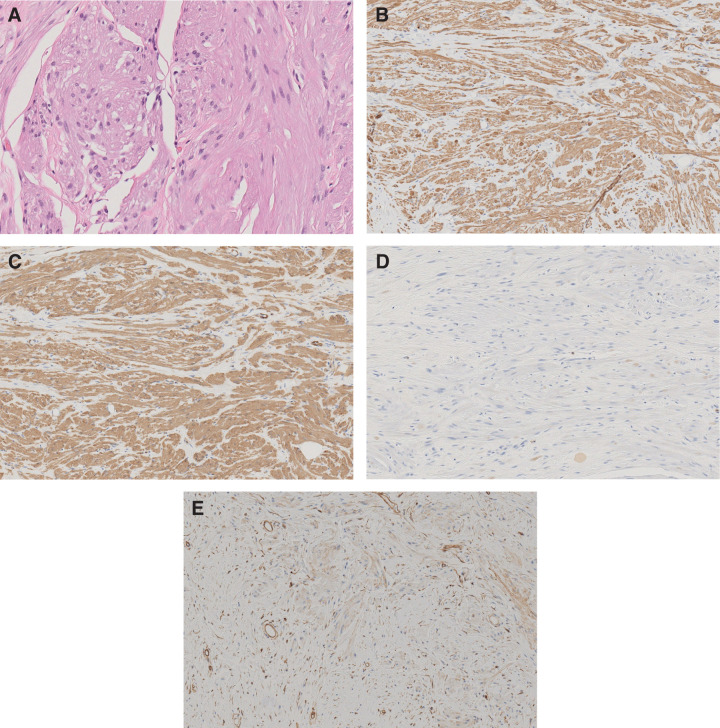
(**A**) Hematoxylin–eosin staining showing a proliferation of smooth muscle cells (×20). (**B**) Positive desmin staining in the epithelial cells (×20). (**C**) α-SMA staining in the epithelial cells (×10). (**D**) Negative S100 staining in the epithelial cells (×10). (**E**) Positive or CD34 staining in the epithelial cells (×10). α-SMA, alpha-smooth muscle actin; CD34, cluster of differentiation 34

Histopathological examination revealed proliferation of spindle-shaped smooth muscle cells. Immunohistochemistry was positive for desmin and alpha-smooth muscle actin (αSMA), partially positive for cluster of differentiation 34 (CD 34), and negative for S100, consistent with a diagnosis of leiomyoma (**Fig. 6**). The operative time was 86 minutes, with an estimated blood loss of 1 mL.

The postoperative course was uneventful, and the patient was discharged on POD 2. Pelvic US showed no evidence of uterine fibroids.

The initial symptoms of chest pain and cough had resolved by the time of the first outpatient visit, and their relevance to the mediastinal tumor remains uncertain.

## DISCUSSION

Two key findings emerged from this case. First, leiomyomas can arise infrequently in the anterior mediastinum and may require surgical diagnosis and treatment. Second, the confronting upside-down VATS approach proved useful for anterior mediastinal tumors.

Primary mediastinal leiomyomas are extremely rare and thought to originate predominantly from the esophagus or mediastinal vascular structures. Possible histogenetic origins include smooth muscle cells of the vascular walls and mesenchymal progenitor cells with the potential for smooth muscle differentiation.^11)^ In this case, the tumor was in close continuity with the pericardial fat tissue in the anterior mediastinum. While the adipose tissue itself is unlikely to be the direct origin, the possibility of smooth muscle proliferation within the fat tissue cannot be ruled out due to the lack of definitive histopathological analysis.

Although metastasizing uterine leiomyoma can involve the mediastinum,^15)^ no evidence of uterine leiomyoma was identified in this patient, supporting the diagnosis of a primary mediastinal leiomyoma.

Due to their rarity and lack of distinctive imaging features, differentiation of mediastinal leiomyomas from other anterior mediastinal tumors is challenging.^8,16–18)^ Therefore, definitive diagnosis requires pathological confirmation through surgical resection.

Mediastinal leiomyomas are typically asymptomatic and discovered incidentally, but may become symptomatic with progressive tumor growth compression of adjacent mediastinal structures. Therefore, complete surgical resection is recommended both for definitive diagnosis and to prevent symptom progression.

For anterior mediastinal tumors, VATS is often performed using the conventional “look-up” method, in which the camera port is typically inserted at lower ICSs, such as the 7th or 8th. However, this approach is prone to interference from the diaphragm, particularly when dealing with lesions located nearby. By contrast, the confronting upside-down VATS technique involves inserting the thoracoscope through higher ICSs (e.g., the 3rd to 5th), allowing for a clear operative view from the apex to the base of the thoracic cavity. Moreover, rotating the assistant’s monitor 180 degrees allows both the surgeon and the assistant to share a visual field similar to that in open thoracotomy, facilitating better coordination and spatial orientation. Separate monitors, with one positioned upside-down, for the surgeon and the assistant, resolve the mirror-image confusion, allowing intuitive maneuvering akin to a conventional thoracotomy. Furthermore, compared with the look-up VATS approach—where instruments may interfere with each other in areas distant from the camera port—the confronting upside-down VATS approach allows direct visualization of the tips of all instruments, enhancing surgical precision and reducing the risk of collisions.^19)^

While this technique has primarily been applied in lung cancer surgery, our experience suggests that recently it also offers significant advantages in mediastinal tumor resection^20,21)^ While the lesion of the present case was located adjacent to the phrenic nerve and close to the diaphragm, this approach enabled a stable surgical field without diaphragmatic interference. Furthermore, precise manipulation allowed to safely preserve the entire course of the phrenic nerve. In the present case, potentially malignant tumors such as solitary fibrous tumor were included in the differential diagnosis; therefore, en bloc resection with an adequate surgical margin, including the surrounding pericardial fat tissue, was required. With the conventional look-up VATS approach, visualization from a caudal perspective may have made identification and preservation of the cranial course of the phrenic nerve technically challenging. In addition, because the lesion was located relatively low in the mediastinum, insertion of the camera through a lower intercostal space would likely have increased the risk of diaphragmatic interference.

The confronting upside-down VATS setting effectively addressed these concerns by providing a stable operative field with a favorable viewing angle, thereby facilitating precise dissection and safe preservation of the phrenic nerve. However, the concomitant use of CO_2_ insufflation might have further improved exposure and maneuverability, which remains a subject for future investigation.

## CONCLUSIONS

Primary leiomyomas can arise infrequently in the anterior mediastinum. Due to their nonspecific imaging findings, distinguishing them from other anterior mediastinal tumors is often difficult, necessitating surgical resection both for definitive diagnosis and treatment. The confronting upside-down VATS approach offers excellent visualization and maneuverability, and may be particularly useful for anterior mediastinal tumors in proximity to the diaphragm.

## References

[1] Roden AC, Fang W, Shen Y, et al. Distribution of mediastinal lesions across multi-institutional, international, radiology databases. J Thorac Oncol 2020; 15: 568–79. 31870881 10.1016/j.jtho.2019.12.108

[2] Takeda S, Miyoshi S, Akashi A, et al. Clinical spectrum of primary mediastinal tumors: a comparison of adult and pediatric populations at a single Japanese institution. J Surg Oncol 2003; 83: 24–30. 12722093 10.1002/jso.10231

[3] den Bakker MA, Marx A, Mukai K, et al. Mesenchymal tumours of the mediastinum—part II. Virchows Arch 2015; 467: 501–17. 26358060 10.1007/s00428-015-1832-6 PMC4656710

[4] Yazici U, Gülhan E, Yazici U, et al. A case of giant mediastinal leiomyoma. Turk J Gastroenterol 2011; 22: 656–7. 22287421

[5] Rajasingham AS, Cooray GH. A large leiomyoma of the mediastinum. Br J Surg 1954; 41: 446–7. 13126499 10.1002/bjs.18004116833

[6] Uno A, Sakurai M, Onuma K, et al. A case of giant mediastinal leiomyoma with long-term survival. Tohoku J Exp Med 1988; 156: 1–6. 10.1620/tjem.156.1 3057681

[7] Ouadnouni Y, Achir A, Bekarsabein S, et al. Primary mediastinal leiomyoma: a case report. Cases J 2009; 2: 8555. 19830084 10.4076/1757-1626-2-8555 PMC2740108

[8] Levesque MH, Aisagbonhi O, Digumarthy S, et al. Primary paratracheal leiomyoma: increased preoperative diagnostic specificity with magnetic resonance imaging. Ann Thorac Surg 2016; 102: e151–4. 27449453 10.1016/j.athoracsur.2016.01.015

[9] Asaf BB, Bishnoi S, Puri HV, et al. Robotic enucleation of oesophageal leiomyoma technique and surgical outcomes. J Minim Access Surg 2022; 18: 84–9. 35017397 10.4103/jmas.JMAS_263_20 PMC8830568

[10] Khalaileh A, Savetsky I, Adileh M, et al. Robotic-assisted enucleation of a large lower esophageal leiomyoma and review of literature. Int J Med Robot 2013; 9: 253–7. 23401224 10.1002/rcs.1484

[11] Haratake N, Shoji F, Kozuma Y, et al. Giant leiomyoma arising from the mediastinal pleura: a case report. Ann Thorac Cardiovasc Surg 2017; 23: 153–6. 27885214 10.5761/atcs.cr.16-00137 PMC5483863

[12] Hakeem ZA, Rathore SS, Wahid A. Rare mediastinal leiomyoma in a child. Gen Thorac Cardiovasc Surg 2017; 65: 415–7. 27640051 10.1007/s11748-016-0705-5

[13] Kim YD, Jeong CS, Jeon WH, et al. Successful thoracoscopic resection of a large mediastinal angiomyolipoma. J Thorac Dis 2017; 9: E427–31. 28616301 10.21037/jtd.2017.04.58 PMC5465168

[14] Marshall MB, DeMarchi L, Emerson DA, et al. Video-assisted thoracoscopic surgery for complex mediastinal mass resections. Ann Cardiothorac Surg 2015; 4: 509–18. 26693146 10.3978/j.issn.2225-319X.2015.11.01 PMC4669254

[15] Abramson S, Gilkeson RC, Goldstein JD, et al. Benign metastasizing leiomyoma: clinical, imaging, and pathologic correlation. AJR Am J Roentgenol 2001; 176: 1409–13. 11373202 10.2214/ajr.176.6.1761409

[16] Li C, Lin F, Pu Q, et al. Primary mediastinal leiomyoma: a rare case report and literature review. J Thorac Dis 2018; 10: E116–9. 29607199 10.21037/jtd.2018.01.37 PMC5864695

[17] Yang PS, Lee KS, Lee SJ, et al. Esophageal leiomyoma: radiologic findings in 12 patients. Korean J Radiol 2001; 2: 132–7. 11752983 10.3348/kjr.2001.2.3.132 PMC2718110

[18] Rodríguez E, Pombo F, Aguilera C, et al. Recurring tracheal leiomyoma presenting as a calcified mediastinal mass. Eur J Radiol 1996; 23: 82–4. 8872076 10.1016/0720-048x(95)00718-6

[19] Mun M, Ichinose J, Matsuura Y, et al. Video-assisted thoracoscopic surgery lobectomy via confronting upside-down monitor setting. J Vis Surg 2017; 3: 129. 29078689 10.21037/jovs.2017.07.08 PMC5638999

[20] Kambe M, Oki T, Iizuka S, et al. Confronting upside-down video-assisted thoracic surgery approach for hemorrhagic bronchogenic cyst manifested by sudden back pain. Surg Case Rep 2025; 11: 24-0126. 10.70352/scrj.cr.24-0126 PMC11861584 40012964

[21] Ueda E, Oki T, Iizuka S, et al. Delayed hemorrhage following needle aspiration for a mediastinal cyst: the significance of confronting upside-down video-assisted thoracic surgery under two-lung ventilation. Surg Case Rep 2025; 11: 25-0134. 10.70352/scrj.cr.25-0134 PMC12056370 40337546

